# Supplementary material for: Impact of Vaccinating Adult Women Who Are HPV-Positive or with Confirmed Cervical SIL with the 9-Valent Vaccine—A Systematic Review
Source: Viruses. 2025 Oct 15;17(10):1377. doi: 10.3390/v17101377 (PMC12568279; doi:10.3390/v17101377)
Supplement: Supplementary file 1 [file viruses-17-01377-s001.zip › viruses-3858195-supplementary.pdf]

Table S1- PICOS

Eligibility Criteria (PICOS)

- **Population:** Adult women (≥18 years) who are HPV-positive or have histologically confirmed SIL.
- **Intervention:** 9-valent HPV vaccine (Gardasil 9), administered before, during, or after treatment.
- **Comparison:** Unvaccinated HPV-positive women; different timings of vaccination.
- **Outcomes:** Viral clearance, SIL regression, CIN2+ recurrence, progression to higher-grade disease, and treatment-related outcomes.
- **Study Design:** Prospective or retrospective cohort studies; observational studies.
- **Exclusion:** Children, men, HIV-positive or immunocompromised populations, non-English studies, reviews, meta-analyses, case reports, letters.

Search Strategy

- Databases: MEDLINE (PubMed), Scopus, Cochrane Library.
- Search date: 7 July 2025.
- Terms: "HPV vaccination OR 9-valent vaccine OR Gardasil 9" AND "HPV positive OR SIL OR HSIL OR after treatment" AND "women."
- Manual reference screening included.
